# Therapeutic angiogenesis in cardiovascular disease

**DOI:** 10.1186/1749-8090-2-49

**Published:** 2007-11-16

**Authors:** Hilal Al Sabti

**Affiliations:** 1 Department of surgery, Sultan Qaboos University Hospital, Code 123. P.Box 35, Al Khod, Sultanate of Oman

## Abstract

Atherosclerotic disease of the arteries is a major cause of coronary artery disease, peripheral vascular disease and stroke. Some patients are however not candidate for the standard treatment of angioplasty or bypass surgery. Hence there is tremendous enthusiasm for the utilization of angiogenesis as a therapeutic modality for atherosclerotic arterial disease. This augmentation of physiological neo-vascularization in cardiovascular disease can be achieved through different pathways. In this article we are reviewing the Use of Gene therapy, Protein therapy and cellular therapy.

## Introduction

In the 20^th ^century, the number of patients affected by atherosclerosis increased dramatically. Cardiovascular disease due to atherosclerosis had become the first cause of death worldwide as compared to infectious diseases at the beginning of the century. The natural history of atherosclerosis can be divided into three stages:

• Stage I: Asymptomatic fatty streak and fibrous plaque formation.

• Stage II: Symptomatic: Formation of fibrous plaque with medial calcification; enlargement of the fibrous plaque and thrombus formation; significant homodynamic occlusion of the vessel and then complete occlusion.

• Stage III: Where three major complications are seen:

- Myocardial infarction.

- Brain infarction.

- Gangrene.

### Risk factors and disease management

The risk factors for having atherosclerotic disease are age, hyperlipidemia, smoking, hypertension, physical inactivity, obesity and hormone status. The conventional management of atherosclerotic arterial insufficiency can be divided into three major parts:

• Risk factors modification and medical treatment in order to restore the balance between the oxygen demand and oxygen supply and prevention of acute thrombotic episodes, these can be achieved by: Lifestyle modification (exercise, smoking cessation, weight reduction), and Cholesterol lowering. Another risk factor modification is Blood pressure management through the following therapies like Aspirin therapy, Beta- blocker, ACE inhibitors. And the last is through interventional therapy which is divided into: Surgical: Includes aorto-coronary bypass, transmyocardial revascularization (TMR) and peripheral vascular bypass and catheter-based: which include percutanous trans-coronary angioplasty and stenting or coronary atherectomy.

Despite the improvement of these interventions and outcomes there are many patients that are not suitable candidates because of their diffuse and severe atherosclerotic CAD and/or because of the risky nature of the intervention. Hence the idea came of promoting and stimulating the process of angiogenesis with newly created vessels which could serve as a biological bypass of the atherosclerotic vessel.

### Neovascularization

During the development of the embryo and the physio-logical repair of any tissue damage, the formation of new blood vessels plays a major role [1]. Some of this new blood vessel formation is normal and beneficial as can be seen in wound healing after trauma, ischemic tissue restoration and in the endometrium during the menstrual cycle. Abnormal formation of blood vessels however, also occur and contributes to a number of important disease processes such as diabetic retinopathy, malignancy, and diseases like rheumatoid arthritis, systemic lupus erythematosus and in atherosclerotic disease[2-5]. Stimulation of new blood vessel growth must therefore be site specific and organ specific.

There are three major processes that contribute to the growth of new blood vessels that are termed globally as neovascularization:

• **Vasculogenesis**: At the beginning it was thought that this happens during embryogenesis in which there is a differentiation of embryonic mesenchymal cells (such as the endothelial precursor cells or angioblasts) into endothelial cells and the de novo development of blood vessels but now it is well known that it contribute to the adult neovasculirization as well [6]. This process is believed to be induced by FGF, VEGF and other angiogenic factors or/and tissue ischemia. This process leads to the development of new blood vessels that are formed are composed mainly of endothelial cells and are referred to as the capillary plexus. The smooth muscle layer that incorporates in the plexus results from migration and proliferation of different cell types like pericytes, smooth muscles cells and fibroblasts [7,8].

• **Angiogenesis**: It is the formation of new blood vessels by sprouting from pre existing small vessels in adult and embryonic tissue or by intravascular subdivision process called intussusception [9,10]. These new blood vessels are usually lacking developed media. It occurs as the level of local angiogenic stimuli increases; this will lead to the activation of the endothelial cells found in pre-existing vessels, with subsequent vasodilatation, increase permeability and the disruption of the basement membrane encompassing endothelial cells of the pre-existing capillaries via proteolytic degradation. Disturbance of the basement membrane allow the cytoplasmic process to extend from the activated endothelial cells, directing their migration and sprouting to the extra vascular space towards the angiogenic stimuli [11]. The cells elongate and aligned to form capillary sprouts and then these sprouts will form a lumen and connect with the neighboring vessels, these capillaries are very small in size and their contribution to the actual blood flow with no or little significance to the ischemic tissue.

• **Arteriogenesis**: It is defined as a rapid proliferation of pre-existing collateral vessels and these vessels have fully developed tunica media [9,12]. It is a mature type of neovascularization which can restore perfusion to an ischemic area.

The occurrence of both angiogenesis and arteriogenesis has been shown in many animal models and in patients with coronary artery disease. Tissue ischemia is one of the key factors that stimulate neovascularization [9].

Folkman has described a series of morphological and biochemical events involved in angiogenesis as, Vasodilatation of parent vessel, degradation of basement membrane, endothelial migration, endothelial cell proliferation, lumen formation, loop formation, formation of new basement membrane, and incorporation of pericytes [13].

### Angiogenesis triggering factors

It is a very complex process. Conceptually the major triggers of this process can be simplified into three broad categories: mechanical, chemical and molecular factors.

#### 1. Mechanical influences

and this can by explained by two factors either through homodynamic or the shear stress:

• *Hemodynamic*: Augmentation of blood flow during exercise, the hyperthyroid state (Thyrotoxicosis) and administration of certain drugs have all been shown to stimulate vascular sprouting[14-17]. It also has been shown that large vessels with low flow tend to reduce or obliterate their endovascular diameter and the lumens of the small vessels with chronically increased high blood flow tend to enlarge [18]. Augmentation of blood flow therefore may both stimulate vascular sprouting and maintain patency of the newly formed collateral vessels and so providing blood flow to the ischemic area.

• *Shear Stress*: Shear stress has an important influence on the development of collaterals in the ischemic tissues. Shear stress and stretch of the myocardium (i.e. increase left ventricular end diastolic volume) can lead to up-regulation of adhesion molecules in the endothelium, attraction of the inflammatory cells and stimulation of the endothelial cells to produce angiogenic factors[19-22].

#### 2. Chemical influence

this can happen either through hypoxia or through the production of nitric oxide:

• **Hypoxia and oxygen tension gradient**: Hypoxia stimulates macrophages to release various factors including platelets derived growth factor and fibroblast growth factor 1 and 2 [23]. Hypoxia has been shown to upregulate vascular endothelial growth factor owing both by increasing transcription mediated by hypoxia inducible factor 1 and by increasing the stability of vascular endothelial growth factor mRNA [24-26]. Hypoxia also increase the expression of vascular endothelial growth factor receptors(FLK and FLT) this renders the endothelium susceptible to either the systematic or the paracrine effect of pre-existing vascular endothelial growth factor [27,28].

• **Nitric oxide**: VEGF is known to induce the release of Nitric oxide from endothelial cells, and vascular endothelium inducible NO synthase (iNOS) production is amplified during VEGF- induced angiogenesis [28]. The role of Nitric Oxide in VEGF induced angiogenesis have been shown in NOS knocked out mice as well as after NOS inhibition, both result in reduction of angiogenesis [29-32]. Nitric Oxide has also a regulatory effect on VEGF production.

#### 3. Molecular influences

• **Effect of glucose on VEGF expression**: Hypoglycemia increases VEGF expression, and after equilibration of the glucose concentration the level of VEGF return back to normal [33,34]. This most likely happen when intracellular CA^2+ ^levels increases in glucose deprived environment leads to activation of protein kinase C. this process induces the activation of Angiopoiten -1, leading to an increase of VEGF expression [35]. Not only hypoglycemia increases VEGF expression but also hyperglycemia do the same thing both of which can result an increase in the production of VEGF[36,37].

• **Inflammation**: Animal studies have shown that the presence of inflammatory cells, like macrophages and neutrophils is sufficient to produce angiogenesis. Following myocardial necrosis, the influx of inflammatory cells including macrophages, monocytes, and platelets results in the release of cytokines that are capable of stimulating fibroblast growth factor and vascular endothelial growth factor expression [38,39]. Vascular endothelial growth factor by itself can stimulate and recruit other macrophages to stimulate inflammation response and further stimulate angiogenesis.

• **Angiogenic growth factors**: The existence of angiogenic factors were first observed with the isolation of tumor factors that generate mitogenic activities in endothelial cells [40-42]. They are induced by a many varieties of cells and their functions are development as well as tumor angiogenesis. Angiogenic growth factors are called so because of their ability to induce the proliferation of various cells in vitro, which contribute to the process of angiogenesis in vivo as shown by studies of animal models. We will describe briefly couple of these growth factors:

**a. Fibroblast growth factor(FGF)**: The first angiogenic growth factor to be discovered as a member of a family currently comprises at least 20 molecules with extensive mitogenic potentials representing some of the most potent angiogenic peptide [41-43]. They are produced by vascular endothelial cell and smooth muscle cells. One characteristic of the FGF family is the ability to interact with heparin- like glycosaminoglycan of the extracellular matrix [44]. The two most widely researched isoforms are FGF-1 and FGF-2[45-48].

**b. Vascular endothelial growth factor (VEGF)**: Was initially purified as vascular permeability factor from tumor cell ascites [49]. It is now known as a multifunctional peptide capable of inducing receptor- mediated endothelial cell proliferation and angiogenesis both in vivo and in vitro. It has a crucial role in embryonic vascular development and in adult pathophysiology. VEGF is a family composed of five members and their effect mediated through three receptors (VEGFR)[50-53].

**c. Placenta growth factor**: Got its name because of it predominance in placental tissue. It has little angiogenic effect in vitro but it seems that both PLGF and VEGF maybe co expressed in vivo [54-56].

**d. Angiopoietin**: is a recently discovered family of growth factors and angiopoietin1 (Ang1) is expressed in tissue close to the blood vessels, suggesting a paracrine mode of action. Angiopoietin 2 (ANG2) is found at sites of tissue remodeling [57-60].

**e. VEGF receptors**: In humans, the effect of VEGF on endothelial cells is mediated via two membrane spanning receptors, VEGFR1 and VEGFR2. Both receptors have high affinity for VEGF [61-63]. VEGFR1 Promotes cell migration, paly a role in organization of blood vessels and gene expression of monocytes and macrophages. While VEGFR2 play a major role in mitogenesis, differentiation of endothelail cells, promotion of cell migration and enhancement of vascular permeability.

## Therapeutic angiogenesis

*Therapeutic angiogenesis *is the clinical use of methods to enhance or promote the development of collateral blood vessels in ischemic tissue. Angiogenic treatment is potentially important as we see more patients that are having ischmia or vascular insufficiency that are not amenable to revascularization. This new form of treatment is an alternative to high risk percutaneous coronary intervention or coronary artery bypass surgery. It can also be used in combination with surgery to provide more complete revascularization.

The ideal agent for therapeutic angiogenesis should have a potent effect, with sustained clinical benefit [64]. It should be specific for the targeted ischemic tissue, with no chronic side effects. It should maintain a high local concentration and adequate exposure time. It should be that the ability of readministration feasible and non invasive way of delivering the agent should be considered.

There are three major ways to promote angiogenesis: protein therapy, gene therapy and cellular therapy.

### 1. Protein therapy

angiogenesis can be achieved by growth factor proteins. These factors include VEGF, bFGF through different routes [65]. There are conflicting results reported in the literature as to the efficacy of these agents [66]. The argument in favor of protein therapy are Finite temporal exposure to angiogenic factor, titratable dosing of angiogenic factor exposure, no exposure to exogenous genetic material, no exposure to viral vector, readministration may be easier; decreased risks of inflammatory response or immune inactivation at the time of repeat dosing, modulation of protein structure and/or combination with slow- release delivery system may abrogate issue of protein stability.

The argument against Protein Therapy is that these proteins have short serum half life and so tissue half life and that would lead to the need of frequent administration and higher doses.

### 2. Gene therapy

Gene therapy is known as any manipulation of gene expression or function for the treatment of specific disease [66-68]. The advantages of gene therapy are, sustained production of angiogenic factor resulting in prolonged exposure to elevated level, the ability for local delivery and so less systemic exposure. This all can be done with one dose as well and the production of angiogenic factor can be directed to specific cell type and so less side effects.

The disadvantages for gene therapy include:

• Introduction of foreign genetic material.

• Exposure to viral vectors with concomitant risk of inflammatory response, viral persistence, and in vivo recombination.

• Potential for non- targeted cell gene delivery.

• Inability to precisely modulate gene expression and thus angiogenic factor levels with present vector system.

• Potential for inactivation and/or inflammatory response at readministration.

• Potential for long term, low level systemic exposure to secreted angiogenic factors.

• Accelerated atherosclerosis [69,70].

• Vascular malformation in the sense of hemangioma formation[71-73]

• Neoplasm: there is a raise in concern regarding stimulating carcinogenesis[41].

• Retinopathy [74,75].

• Edema: It has been shown that the presence of high VEGF levels lead to augmentation of vascular permeability [76,77].

### 3. Cellular therapy

this is a new approach in which cells that are known to produce angiogenic factors are used to promote angiogenesis in ischemic tissue [78]. Many cell types have been used for this purpose as monocytes and endothelial progenator cells and marrow stromal cells. These cells have been shown to stimulate angiogenesis through expression of growth factors stimulated by local paracrine stimuli from ischemia. These cells can also participate as cellular precursors for vasculogenesis and they can act as a vehicle for delivery of therapeutic genes coding for angiogenic factors.

## Clinical trials in therapeutic angiogenesis

The results from the preclinical studies about the growing new blood vessels in the ischemic areas in the heart and the peripheral parts of the body had led to several clinical trials which are mainly phase 1 trials investigating the efficacy of different routs of delivery of protein, gene or cellular therapy.

Few studies were carried out by injecting VEGF protein through intracoronary and intravenous routs in different dose scales and showed improvement in symptoms and in perfusion [79,80]. The problem came with it was the results were dose dependent and hypotension was quite a clear complication. The VIVA study was a randomized double blinded, placebo controlled study that showed no difference between the two groups in improvement of symptoms, quality of life And exercise tolerance [81]. The FGF-2 protein was also studied using intracoronary and intravenous route in no option patients. Laham et al had studied in fifty two patients and they received intracoronary protein and there was a significant improvement in quality of life and exercise time and the infracted defect size, but the control group was lacking in this study [82]. The FIRST trial was a multicentric randomized double blinded placebo controlled trial and was designed to examine the safety, pharmacokinetics and efficacy of FGF-2 protein but did not show any improvement in exercise tolerance or myocardial perfusion.

As for the gene therapy, few studies with small numbers of patients were published till the Angiogenic Gene Therapy trial (AGENT) with intracoronary infusion of adenovirus encoding FGF-2. This study stopped enrollment when the interim analysis showed no possibility of detected efficacy. even though the subgroup analysis with the exercise tolerance test of less then 10 minutes did show an improvement [83].

For the cellular therapy, a number of clinical trials for the endothelial progenitor cells therapy were published in the last few years. Most of those studies had similar results which showed a 7–9% improvement in global left ventricular function and remodeling of left ventricle [84]. Other studies for the intramyocardial percutanouse delivery of autologus bone marrow had shown that this procedure is safe and it improved Angina symptoms and improve myocardial perfusion and increase left ventricular function[85-87]. Long term studies are not available.

## Conclusion

Therapeutic angiogenesis seems to be the promising hope for patients with advanced vascular diseases not responding to the conventional treatment. The different methods that have been approached in the last two decades keep us with hope and caution. Couscous measures have to be taken into consideration in applying any the treatment options as the unwanted side effects can be prominent. The present trials in the literature right now do not have the full answer and more trials will be needed in order to document these methods as routine alterative methods. Till then we should stick with our conventional methodology whenever it is feasible.

## Authors' contributions

HA: Acquisition of data, design of audit, Literature search and preparation of draft of manuscript
